# SSTR2A expression in medullary thyroid carcinoma is correlated with longer survival

**DOI:** 10.1007/s12020-018-1706-1

**Published:** 2018-08-20

**Authors:** Lisa H. de Vries, Lutske Lodewijk, Stefan M. Willems, Koen M. A. Dreijerink, Bart de Keizer, Paul J. van Diest, Abbey Schepers, Han J. Bonenkamp, Ilse A. C. H. van Engen-van Grunsven, Schelto Kruijff, Bettien M. van Hemel, Thera P. Links, Els J. M. Nieveen van Dijkum, Susanne van Eeden, Gerlof D. Valk, Inne H. M. Borel Rinkes, Menno R. Vriens

**Affiliations:** 1 Department of Surgery, University Medical Centre Utrecht, Heidelberglaan 100, 3584CX Utrecht, The Netherlands; 2 Department of Pathology, University Medical Centre Utrecht, Heidelberglaan 100, 3584CX Utrecht, The Netherlands; 3 Department of Endocrine Oncology, University Medical Centre Utrecht, Heidelberglaan 100, 3584CX Utrecht, The Netherlands; 4 Department of Radiology, University Medical Centre Utrecht, Heidelberglaan 100, 3584CX Utrecht, The Netherlands; 5 Department of Surgery, Leiden University Medical Centre, Albinusdreef 2, 2333ZA Leiden, The Netherlands; 6 Department of Surgery, Radboud University Medical Centre, Geert Grooteplein 8, 6525GA Nijmegen, The Netherlands; 7 Department of Pathology, Radboud University Medical Centre, Geert Grooteplein 8, 6525GA Nijmegen, The Netherlands; 8 Department of Surgery, University Medical Centre Groningen, Hanzeplein 1, 9700RB Groningen, The Netherlands; 9 Department of Pathology, University Medical Centre Groningen, Hanzeplein 1, 9700RB Groningen, The Netherlands; 10 Department of Internal Medicine, University Medical Centre Groningen, Hanzeplein 1, 9700RB Groningen, The Netherlands; 11 Department of Surgery, Academic Medical Centre Amsterdam, Meibergdreef 9, 1105AZ Amsterdam, The Netherlands; 12 Department of Pathology, Academic Medical Centre Amsterdam, Meibergdreef 9, 1105AZ Amsterdam, The Netherlands

**Keywords:** Medullary thyroid carcinoma, Immunohistochemistry, Somatostatin receptor 2A, Tissue microarray, Oncology

## Abstract

**Purpose:**

Medullary thyroid carcinoma (MTC) derives from the parafollicular C-cells of the thyroid gland. Somatostatin receptors (SSTRs) are expressed in various neuroendocrine tumours including MTC. The aim of this study was to evaluate SSTR2A as a prognostic factor for MTC, to study distribution of SSTR2A expression within tumours and to compare expression of SSTR2A between primary tumours and corresponding lymph node metastases.

**Methods:**

Patients who underwent surgery between 1988 and 2014 for MTC from five tertiary referral centres in The Netherlands were included. In total, primary tumours of 114 patients and lymph node metastases of 34 patients were analysed for expression of SSTR2A using a tissue microarray, and correlated with clinicopathological variables and survival.

**Results:**

The mean age of patients was 45.5 years (SD 16.2), 55 patients were male (49.5%). Primary tumours of 58 patients (50.9%) showed SSTR2A expression. In multivariate Cox-regression analysis, SSTR2A positivity correlated independently with better overall survival (OS) (HR 0.3; 95% CI 0.1–1.0). In stage IV MTC patients, 10-year survival rates for SSTR2A-negative and positive patients were 43% and 96%, respectively. In 53.9% of patients with lymph node metastases, expression in primary tumour and lymph node metastases differed.

**Conclusion:**

SSTR2A expression is correlated with longer OS in MTC, especially for stage IV patients, suggesting that SSTR2A expression might be a useful prognostic factor in MTC. The SSTR2A status of the primary MTC does not predict expression in lymph node metastases.

## Introduction

Medullary thyroid carcinoma (MTC) is derived from the parafollicular clear cells (C-cells) of the thyroid gland [1]. Although MTC accounts for only 4% of all thyroid carcinomas, it is accountable for about 13% of deaths resulting from thyroid cancer [2]. Besides a sporadic form that occurs in the majority of cases, MTC develops in the remaining 25–30% of patients as part of the hereditary tumour syndrome Multiple Endocrine Neoplasia type 2 (MEN2) [1]. Patients who are treated with curative intent should always have a total thyroidectomy and dissection of the central lymph node compartment. Adjuvant therapy in general is of limited value [3–5]. Unfortunately, in more than 50% of patients, calcitonin levels remain elevated after supposed curative resection, indicating that subclinical active tumour tissue is still present [3, 6].

Early diagnosis of MTC is very important to improve prognosis. When diagnosed in an early phase (stage I and II), 10-year survival rates are favourable at 100% and 93%, respectively. Unfortunately, approximately 45% of patients present with metastases (stage III and IV), associated with 10-year survival rates of 71% and 21%, respectively [3]. Prognosis is difficult to predict in stage III or IV MTC, whereas this is essential for personalised follow-up and adjuvant therapy strategies for patients that will rapidly progress [7].

The C-cells of the thyroid gland have the ability to express somatostatin receptors (SSTRs) like many neuroendocrine tumours. There are six different subtypes: SSTR1, SSTR2A, SSTR2B, SSTR3, SSTR4 and SSTR5 [8]. These receptors have a broad spectrum of biological actions including inhibition of proliferation, cell-survival and angiogenesis [8–10]. In this study, we focused on SSTR2A since this particular receptor is used as a target for nuclear imaging as well as for medical therapy. Expression of SSTR2 enables the use of somatostatin analogues (SSTA) for therapeutic purposes, which has a positive effect on the symptoms accompanying MTC like diarrhoea, weight loss and malaise [11–13]. Unfortunately SSTA therapy does not reduce tumour mass, nor improve survival rates [12]. Peptide receptor radionuclide therapy (PRRT) with radioactively labelled SSTA shows promising results in other endocrine tumours, with much higher response rates than SSTA therapy. In a recent randomised phase 3 trial in patients with progressive midgut neuroendocrine tumours, treatment with ^177^Lu-Dotatate resulted in a risk of progression or death that was 79% lower than the risk associated with a high-dose of the SSTA octreotide [14]. Limited information is available on the effect of PRRT with SSTA in MTC. Only a few, small sample sized studies have been conducted, but with promising results. Disease progression, stable disease and (partial) response was seen in 14–65%, 0–61% and 18–67% of patients, respectively. Toxicity was low and quality of life improved using PRRT [15–18]. To determine whether a patient is eligible for treatment with PRRT, the receptor subtype and the amount of expression in this specific tumour needs to be established [15, 18]. This can be done by imaging using radioactive SSTA or by immunohistochemistry on tumour tissue [11].

Currently, imaging modalities still have limitations in identifying metastases, resulting in a low specificity and sensitivity [3]. SSTR expression enables the use of SSTA scans for follow-up in patients with persistent MTC for localisation of metastases, especially in the neck lymph nodes [12]. At this moment, stability of SSTR expression between primary tumours and lymph node metastases is not yet investigated in MTC. For the utility of SSTA scans, such information is necessary.

Besides being useful as therapeutic target, SSTR expression might be relevant as a prognostic factor. Herac et al. [8]. studied the correlation between SSTR2A and SSTR5 expression and several clinicopathological features. They demonstrated a significant correlation between SSTR2A expression and the presence of lymph node metastases, which is an indicator of poor prognosis, but no survival analysis was presented [3, 8]. The aim of this study was to evaluate SSTR2A as a prognostic factor in a larger cohort of MTC patients with long-term follow-up. Furthermore, we studied distribution of SSTR2A within tumours and compared expression of SSTR2A between primary tumours and corresponding lymph node metastases.

## Materials and methods

### Patients

Patients who underwent surgery between 1988 and 2014 for MTC were identified from the pathology databases of Leiden University Medical Centre (LUMC), Amsterdam Medical Centre (AMC), Radboud University Medical Centre (RadboudUMC), University Medical Centre Groningen (UMCG) and University Medical Centre Utrecht (UMCU), The Netherlands (all tertiary referral centres). Formalin fixed paraffin embedded (FFPE) tissues were collected from the pathology archives. In total, 114 patients were identified from whom primary tumour tissue was available for inclusion in the tissue microarray (TMA). From two of the five hospitals (LUMC and UMCU), tissue of lymph node metastases was available as well, enabling analysis of lymph node tissue of 34 patients.

Clinical and pathological patient information was retrieved from patient files in all five centres. All MEN2 diagnoses were confirmed by germline mutation analysis of the *RET* gene. Sporadic patients were either patients with negative germline mutation analysis or with a negative family history. Microscopic positive resection margins were considered as part of the T-stage and not included as a separate variable. Disease status was based on postoperative (dichotomous) calcitonin and CEA serum values. No exact values or doubling times were considered since we included patients from five centres over almost three decades for which different assays were applied at the time. An elevation in CEA or calcitonin was interpreted as persistent disease, CEA or calcitonin within normal range was interpreted as cured. Only postoperative CEA and calcitonin values that were measured > 6 months after surgery were taken into account. Whole slides were scored for necrosis, angioinvasion and desmoplasia. Necrosis and angioinvasion were scored as absent or present and desmoplasia as negative, some, moderate or severe. These scorings were performed on the same FFPE blocks that were used for the construction of the TMA.

This study was performed according to national guidelines with respect to the use of leftover tissue and approval for this study, including the use of patient data, was obtained from the Institutional Review Board of the UMCU [7, 19].

### Construction of tissue microarray

The TMA was constructed using an automated machine (TMA grand master, 3D Histec, Budapest, Hungary). Three cores of 0.6 mm were punched from each FFPE block of primary tumour and lymph node metastases if available. To assure that cores were punched from tumour areas, cell rich areas were marked on H&E slides by a pathologist (P.v.D. and L.L.), scanned, and marks were manually circled with the TMA software (3D Histech).

### Immunohistochemistry

The TMA blocks were cut at 4 µm and mounted on coated slides. Staining for SSTR2A was carried out with an automatic immunostainer (Bench Mark ULTRA Automated IHC slide staining system, Ventana Medical Systems, Inc., USA) following protocol: after baking the slides at 75 °C and incubating for eight minutes, the slides were deparaffinised on 72 °C. Antigen retrieval was performed using the CC1 standard pre-treatment. The primary SSTR2A antibody (1:20; code SS-8000-RM(A); rabbit IgG; biotrend) was incubated for 32 min at 37 °C. Slides were counterstained with haematoxylin and coverslipped.

### Scoring of immunohistochemistry

**Fig. 1:**
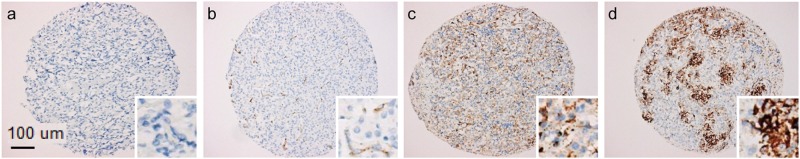
Representative examples of immunohistochemical staining for SSTR2A in TMA of MTC. **a** Absent SSTR2A staining. **b** SSTR2A staining with intensity 1 in 10% of cells. **c** SSTR2A staining with intensity 2 in 100% of cells. **d** SSTR2A staining with intensity 3 in 60% of cells

All cores in the SSTR2A TMA slides were scored by an experienced pathologist (S.M.W.) and an experienced researcher (L.L.) for staining intensity as absent (0), weak (1), moderate (2) or strong (3) and percentage of positive tumour cells. On the basis of Volante et al. [20], we considered only membranous staining positive and discarded cytoplasmic staining. Representative scores of all immunostainings are shown in Fig. 1. Data on hypoxia inducible factor-1 alpha (HIF-1α), vascular endothelial growth factor (VEGF), glucose transporter 1 (Glut-1), carbonic anhydrase IX (CAIX) and microvessel density (MVD) was available from a previous study [7].

### Statistical analysis

Categorical data were summarised with frequencies and percentages, and continuous data were summarised with medians and ranges. To increase the power of the statistical analysis categorical data were recoded into dichotomous variables. Grade of desmoplasia was recoded into none-some and moderate-severe. Stage was recoded into stage I–III and stage IV. Hereditability was recoded in either sporadic disease or MEN2 syndrome [7]. SSTR2A scorings were transformed into a dichotomous variable, being positive when the average intensity of the three cores was ≥ 1, independently of the previously scored percentage of staining.

Overall survival (OS) was defined as the time to death from any cause. Progression-free survival (PFS) was defined as the time to development of distant metastases or death.

Univariate Kaplan–Meier survival analysis was performed and Kaplan–Meier survival curves were plotted. The log-rank test was used to calculate significance. Multivariate Cox-regression analysis was performed and hazard ratios (HR) of clinicopathological characteristics on OS were calculated. All reported *p*-values were two sided. Analysis was performed using SPSS version 24.0 software (SPSS, Inc., Chicago, IL, USA).

## Results

### Clinicopathological variables

The 114 patients included in this study were between eight and 82 years of age (mean age 45.5, SD 16.2). Fifty-five patients were male (49.5%) and 56 (50.5%) were female (in three cases gender was unknown). Sixty patients (57.7%) had sporadic MTC, 40 patients (38.5%) had MEN2a and four patients had MEN2b (3.8%) (in ten patients the mutation status was unknown). Median tumour size ranged from 4 to 70 mm (mean 26.1 mm). At the time of first surgery 68 patients (61.8%) presented with lymph node metastases. Fifteen patients (14.4%) presented with stage I, 25 (24.0%) with stage II, 16 (15.4%) with stage III and 48 (46.2%) with stage IV (in ten patients tumour stage was unknown). Mean follow-up time was 69.5 months (range 0–318 months).

### SSTR2A expression in primary tumours

**Fig. 2:**
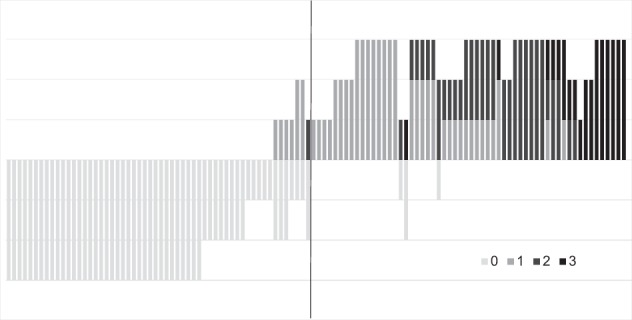
Intensity of all scored cores in each patient individually arranged from low average intensity to high average intensity. Each bar represents one patient. The length of the bar correlates with the amount of cores scored per patient. The colours of the bars represent the intensity of the staining. The patients on the left of the blue stripe are SSTR2A negative, the patients on the right of the blue stripe are SSTR2A positive

Sixty-five patients (57.0%) expressed SSTR2A in their primary tumour, including weak expression (average intensity ≥ 1) in 35 patients (30.7%), moderate expression (average intensity ≥ 2) in 14 patients (12.3%) and strong expression (average intensity 3) in nine patients (7.9%). In the remaining seven patients (6.1%), so little expression (average < 1) was seen that these patients were classified as negative. In 49 patients (43.0%) there was no SSTR2A expression present in the primary tumour. Figure 2 shows the intensity of all scored cores in each patient individually and demonstrates the distribution of different intensities throughout the entire group of patients.

As to primary tumour distribution of SSTR2A expression, ten patients had only one core and were discarded. Of the remaining 104 patients, 72 (69.3%) showed homogeneous expression and 32 (30.8%) showed heterogeneous expression.

### SSTR2A expression in primary tumours vs. lymph node metastases

The average SSTR2A intensity was 0.9 (SD 1.0) of all primary tumours (*n* = 114) and 0.7 (SD 1.1), 0.8 (SD 1.0) and 0.6 (SD 0.7) for lymph node metastases at initial presentation and subsequent surgeries (not significant).

**Fig. 3:**
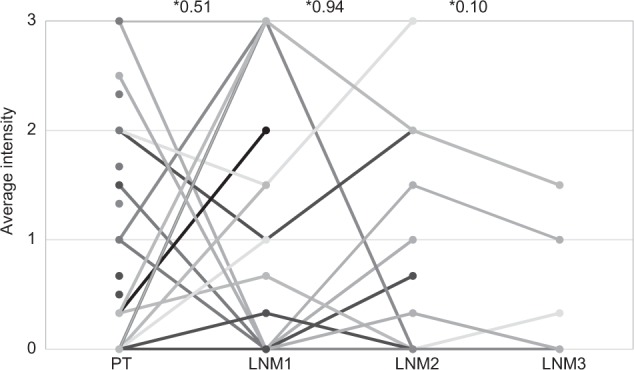
Average intensity of SSTR2A expression in primary tumour and their subsequent lymph node metastases. The vertical axis shows the average intensity of the staining. PT primary tumour, LNM lymph node metastasis. * = *p*-value between average intensities calculated by Wilcoxon Signed Ranks Test

Figure 3 shows the average expression within the various tissues for each individual patient. Five patients (14.7%) showed SSTR2A expression in both primary tumour and all lymph node metastases, but with differences in intensity levels between the primary tumour and lymph node metastases in all five. Eleven patients (32.4%) showed no SSTR2A expression at all in either tissue. Ten patients (29.4%) did not express any SSTR2A in the primary tumour but did show SSTR2A expression in ≥1 of the lymph node metastases. Eight patients (23.5%) showed SSTR2A expression in the primary tumour but no expression in ≥1 of the lymph nodes metastases.

### Association between primary tumour SSTR2A expression and clinicopathological variables

Expression of SSTR2A in comparison with clinicopathological parameters is outlined in Table 1. Heritability (MEN2) and HIF-1α were the only characteristics that were significantly different between SSTR2A-negative and positive groups with 34.1% vs. 65.9%, and 34.5% vs. 65.5%, respectively.

**Table 1:** Clinicopathological characteristics of MTC patients stratified by SSTR2A intensity

	SSTR2A negative		SSTR2A positive		*p*-value
	*N* = 56		*N* = 58		
Mean age in years (SD)	45.3	(16.7)	52	(15.8)	0.90
Gender					1.00
Male (%)	28	(50.9)	27	(49.1)	
Female (%)	28	(50.0)	28	(50)	
Heritability					0.01
Sporadic (%)	38	(63.3)	22	(36.7)	
MEN2a/b (%)	15	(34.1)	29	(65.9)	
Stage					0.56
I–III (%)	28	(51.9)	26	(48.1)	
IV (%)	22	(45.8)	26	(54.2)	
Mean size in mm (SD)	26.8	(14.4)	25.4	(15.7)	0.64
< 20 mm (%)	17	(45.9)	20	(54.1)	0.67
≥ 20 mm (%)	32	(52.5)	29	(47.5)	
Lymph node metastasis					0.08
No (%)	26	(61.9)	16	(38.1)	
Yes (%)	29	(42.6)	39	(57.4)	
Overall survival					0.06
Did not die (%)	38	(44.2)	48	(55.8)	
Died (%)	9	(75.0)	3	(25.0)	
Progression-free survival (%)					0.41
No progression/death	40	(51.3)	38	(48.7)	
Progression/death	12	(46.2)	14	(53.8)	
Disease status					0.15
Normal CEA (%)	14	(37.8)	23	(62.2)	
Elevated CEA (%)	33	(54.1)	28	(45.9)	
Necrosis					1.00
Absent (%)	43	(47.8)	47	(52.2)	
Present (%)	4	(44.4)	5	(55.6)	
Angioinvasion					0.32
Absent (%)	44	(49.4)	45	(50.6)	
Present (%)	3	(30.0)	7	(70.0)	
Desmoplasia					0.23
None-some (%)	27	(54.0)	23	(46.0)	
Moderate-severe (%)	20	(40.8)	29	(59.2)	
HIF-1α					0.00
Negative (%)	30	(68.2)	14	(31.8)	
Positive (%)	20	(34.5)	38	(65.5)	
CAIX					0.43
Negative (%)	28	(52.8)	25	(47.2)	
Positive (%)	21	(43.8)	27	(56.3)	
Glut-1					0.21
Negative (%)	49	(51.0)	47	(49.0)	
Positive (%)	1	(16.7)	5	(83.3)	
MVD (SD)	14.5	(9.5)	13.9	(6.1)	0.72
< 14.3 vessels/core (%)	30	(53.6)	26	(46.4)	0.32
≥ 14.3 vessels/core (%)	19	(42.2)	26	(57.8)	
VEGF					0.84
Negative (%)	17	(45.9)	20	(54.1)	
Positive (%)	30	(49.2)	31	(50.8)	

### Prognostic value

**Fig. 4:**
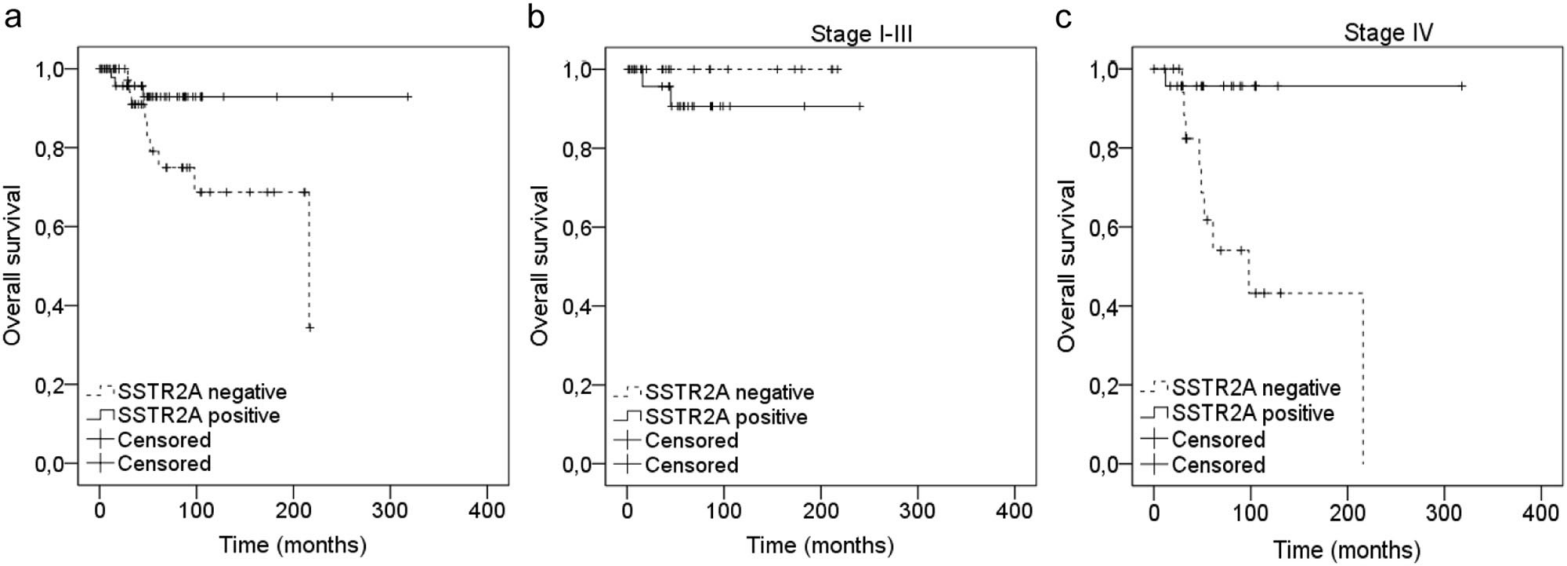
Kaplan–Meier overall survival curves for patients with SSTR2A-positive and negative MTC. **a** Analyses over total group of 114 patients. **b** Analyses over subpopulation of TNM-stage I–III patients. **c** Analyses over subpopulation of TNM-stage IV patients

As outlined in Table 2, for OS a significant prognostic association was found for SSTR2A expression, heritability, stage, lymph node metastases, disease status, necrosis and VEGF. Thirty-eight patients (80.9%) vs. 48 patients (94.1%) survived in the SSTR2A-negative and SSTR2A-positive groups, respectively, (*p* = 0.035). In the Kaplan–Meier curves shown in Fig. 4 the prognostic effect of SSTR2A positivity for all patients is shown, for patients with stage I–III and for patients with stage IV. Within the group of patients having stage IV MTC, 10-year survival rates decrease significantly in SSTR2A-negative patients; from 96% for SSTR2A-positive patients to 43% for SSTR2A-negative patients. In the small subpopulation of patients with lymph node metastases, 10-year survival rates decrease, although not significantly, when SSTR2A is not expressed in ≥1 of the lymph nodes; from 64% for patients with ≥1 positive lymph node to 49% for patients with negative lymph nodes as shown in Supplementary Fig. 1. For PFS a significant association was found for heritability, stage, lymph node metastases, disease status, necrosis, HIF-1α and Glut-1, but not for SSTR2A expression.

**Table 2:** Univariate survival analysis on progression-free (PFS) and overall survival (OS) for MTC patients

	Progression-free survival				Overall survival			
	*N*	PF (*N*)	PF (%)	*p*-value	*N*	OS (*N*)	OS (%)	*p*-value
SSTR2A				0.71				0.04
Negative	52	40	76.9		47	38	80.9	
Positive	52	38	73.1		51	48	94.1	
Gender				0.15				0.48
Male	51	35	68.6		48	41	85.4	
Female	53	43	81.1		50	45	90.0	
Heritability				0.01				0.00
Sporadic	56	39	69.9		52	42	80.8	
MEN2a/b	44	37	84.1		42	41	97.6	
Stage				0.00				0.02
I–III	53	51	96.2		50	48	96.0	
IV	47	23	48.9		45	35	77.8	
Size in mm				0.35				0.87
< 20 mm	36	31	86.1		36	33	91.7	
≥ 20 mm	60	18	70.0		56	49	87.5	
Lymph node metastasis				0.00				0.03
No	40	39	97.5		37	36	97.3	
Yes	64	39	60.9		61	50	82.0	
Disease status				0.00				0.01
Normal CEA/calcitonin	37	35	94.6		37	37	100.0	
Elevated CEA/calcitonin	60	36	60.0		57	45	78.9	
Necrosis				0.00				0.00
Absent	88	68	77.3		85	76	89.4	
Present	9	3	33.3		7	4	57.1	
Angioinvasion				0.11				0.85
Absent	87	66	75.9		83	72	86.7	
Present	10	5	50.0		9	8	88.9	
Desmoplasia				0.33				0.34
None-some	50	40	80.0		48	44	91.7	
Moderate-severe	47	31	66.0		44	36	81.8	
HIF-1α				0.00				0.06
Negative	42	39	90.7		41	39	95.1	
Positive	57	22	61.4		54	44	81.5	
CAIX				0.40				0.25
Negative	53	43	81.1		52	48	92.3	
Positive	46	31	67.4		42	34	81.0	
Glut-1				0.01				0.52
Negative	94	71	75.5		90	78	86.7	
Positive	6	3	50.0		5	5	100.0	
MVD				0.31				0.51
< 14.3 vessels/core	55	37	67.3		51	43	84.3	
≥ 14.3 vessels/core	44	36	81.8		43	39	90.7	
VEGF				0.65				0.03
Negative	37	26	70.3		34	32	94.1	
Positive	59	46	78.0		57	47	82.5

Twelve patients died, which restricted the number of variables we could use in multivariate Cox-regression analysis. We chose to include SSTR2A and stage, because disease stage is currently the strongest prognostic variable available. The variable heritability was left out of multivariate Cox-regression analysis since in our study almost all patients with hereditary tumours had stage I or II MTC. This can be explained by the fact that the majority of these patients had prophylactic thyroidectomies. Lymph node metastasis were not included since this variable is also taken into account when tumour stage is analysed. Disease status was left out because this is predominantly based on postoperative CEA/calcitonin measurements, which only have meaning when evaluated over time, but not as a single value. Since necrosis was only present in 11 patients (7.9%), it is not suitable as a prognostic factor and was, therefore, not included in the multivariate Cox-regression analysis. Desmoplasia, HIF-1α, CAIX, Glut-1 and VEGF were not taken into account since these are all pathological variables while we wanted to see whether SSTR2A expression could add something to the existing clinical prognostic variables.

SSTR2A expression and stage significantly and independently correlated to OS, as outlined in Table 3. SSTR2A expression ≥ 1 decreased the risk to die with a HR of 0.27 (95% confidence interval (CI) 0.07–0.98). Having stage IV, on the other hand, increased the risk of dying with a HR of 5.05 (95% CI 1.10–23.05).

**Table 3:** Multivariate Cox-regression analysis on OS

	Events/patients	Adjusted hazard ratio (95% CI)	*p*-value
SSTR2A			0.05
Negative	9/43	1	
Positive	3/51	0.3 (0.1–1.0)	
Stage			0.04
I–III	2/49	1	
IV	10/45	5.0 (1.1–23.0)	

## Discussion

The aim of this study was to evaluate SSTR2A as a prognostic factor for MTC, to study distribution of SSTR2A expression within tumours and to compare expression of SSTR2A between primary tumours and corresponding lymph node metastases.

We found SSTR2A expression in 50.9% of MTC cases. This is slightly less than the findings of Herac et al. [8] who found SSTR2A expression in 66.0% of patients. Other studies investigated SSTR2 expression in general, not SSTR2A in particular. Zatelli et al. [12] and Papotti et al. [21] studied SSTR2 expression in 51 patients and found positivity in 22 patients (43%). Kendler et al. [22] recently found SSTR2 expression in 12 of 42 studied patients (28.6%). Taken together, approximately half of patients seem to show SSTR2 expression.

In survival analysis, SSTR2A expression was independently associated with better OS, especially in stage IV patients. Therefore, SSTR2A expression might be a valuable prognostic marker in MTC. Interestingly, no association was found for SSTR2A expression and PFS, for which we do not have a good explanation. The more favourable prognosis accompanying SSTR2A expression could be explained by the fact that well differentiated tumours, which still express SSTRs like normal C-cells, tend to have a more indolent course of disease. Herac et al. [8] studied SSTR2A expression in 97 patients with MTC and found that SSTR2A expression could be an adverse prognostic factor since it correlated with presence of lymph node metastases. Herac et al. found that patients with lymph node metastases, were SSTR2A positive in 92.0% and negative in 8.0%. In our study, patients with lymph node metastases were SSTR2A positive and negative in 57.4% and 42.6%, respectively. So, in our study we also found the association between lymph node metastases and SSTR2A expression, however, contrary to Herac et al., in our study this association was not significant. The variance in our results could be explained by the fact that in the study of Herac et al. 25.8% had lymph node metastases at the time of primary surgery, whereas in our study 61.8% of patients presented with lymph node metastases [8].

We show for the first time that in the majority of MTC cases the distribution of SSTRs was homogeneous throughout the primary tumour. This implies that preoperatively identification of the SSTR2A status on core needle or fine needle aspiration biopsies should be possible, which has consequences for therapy strategies such as the use of PRRT. Also in practice, one can imagine that by knowing SSTR2A status of a patient who presents with stage IV disease, the treating physician might be able to give a better indication of prognosis.

Twenty-four percent of cases showed SSTR2A expression in the primary tumour, but not in ≥1 of the lymph node metastases. Furthermore, 29.4% patients who did not show expression in the primary tumour, expressed SSTR2A in ≥1 of the lymph node metastasis. This means that, based on our immunohistochemistry findings, SSTR2A status of the primary tumour is a poor predictor of expression in lymph node metastases, which might indicate that the SSTR2A status of the primary tumour cannot be used as a tool for clinical decision making with regard to SSTR2A nuclear imaging.

Herac et al. [8] found that desmoplasia, CAIX and HIF-1α were significantly correlated with SSTR2A expression. In our study, we did not find significant associations with desmoplasia and CAIX, but could confirm the association with HIF-1α. It is known that somatostatin and octreotide, a SSTR2A-specific SSTA, can block the activation and inhibit the effect of HIF-1α and its downstream targets [23, 24]. This finding suggests a relation between the two factors, even though the correlation is not fully understood yet and this should be investigated on a cellular level.

One of the strengths of our retrospective study is the large sample size, considering MTC has a low incidence. Also, our follow-up time is long (mean 69.5 months, range 0–318 months). Long follow-up is essential since MTC is a tumour with low proliferative activity and low event rates. Furthermore, we used immunohistochemical data, which we were able to combine with clinical endpoints such as the occurrence of distant metastases and death. Limitations of our study mainly are a consequence of the retrospective design of it and the rare occurrence of MTC. In our database, we included patients from five different tertiary referral centres covering almost three decades. Therefore, in our analysis we had to use variables that were consistent over time and between centres. Since we included patients from over almost three decades we have a wide range in our follow-up period. Furthermore, guidelines for surgery have changed over the years and surgical techniques may have differed between centres, which may have resulted in not all patients getting a cervical lymph node resection. This may have caused an underestimation of lymph node-positive patients. Moreover, one might argue that whole slide analysis would be a superior technique to TMA. However, in previous studies, good concordance between TMA and whole slide analysis was found if only one punch was used, with an increasing level of concordance if more punches were considered [25]. Furthermore, for the validation of prognostic biomarkers, TMA is described as the standard [26, 27].

In conclusion, our results show that SSTR2A may be a valuable prognostic biomarker for MTC patients indicating longer OS, especially in stage IV patients. Distribution within the tumour was homogenous in the majority of cases, indicating that preoperative SSTR2A status may be assessed in core needle or fine needle aspiration biopsies. SSTR2A status of the primary MTC is a poor predictor of expression in lymph node metastases.

## Electronic supplementary material

Supplementary figure 1
