# Supplementary figures and images for: SSTR2A expression in medullary thyroid carcinoma is correlated with longer survival
Source: Endocrine. 2018 Aug 20;62(3):639–47. doi: 10.1007/s12020-018-1706-1 (PMC6244936; doi:10.1007/s12020-018-1706-1)

#
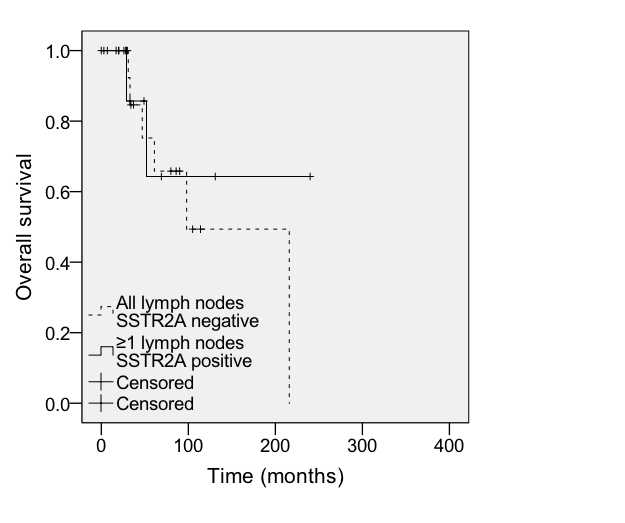

Supplement: Supplementary file 1 — Supplementary figure 1 [file 12020_2018_1706_MOESM1_ESM.docx]
